# Using molecular functional networks to manifest connections between obesity and obesity-related diseases

**DOI:** 10.18632/oncotarget.19490

**Published:** 2017-07-22

**Authors:** Jialiang Yang, Jing Qiu, Kejing Wang, Lijuan Zhu, Jingjing Fan, Deyin Zheng, Xiaodi Meng, Jiasheng Yang, Lihong Peng, Yu Fu, Dahan Zhang, Shouneng Peng, Haiyun Huang, Yi Zhang

**Affiliations:** ^1^ College of Information Engineering, Changsha Medical University, Changsha 410219, P. R. China; ^2^ Department of Mathematics/Network Engineering/Bioscience and Bioengineering/Library, Hebei University of Science and Technology, Shijiazhuang 050018, P. R. China; ^3^ Department of Mathematics, Hangzhou Normal University, Hangzhou 311121, P. R. China; ^4^ Department of Food Science, Fujian Agriculture and Forestry University, Fuzhou 35002, P. R. China; ^5^ Department of Civil and Environmental Engineering, National University of Singapore, Singapore 117576, Singapore; ^6^ Institute of Genetics and Developmental Biology, Chinese Academy of Sciences, Beijing 100101, P. R. China; ^7^ Department of Genetics and Genomic Sciences, Icahn School of Medicine at Mount Sinai, New York, NY 10029, USA

**Keywords:** bioinformatics, human obesity, obesity-related diseases, protein interaction network, gene expression

## Abstract

Obesity is a primary risk factor for many diseases such as certain cancers. In this study, we have developed three algorithms including a random-walk based method OBNet, a shortest-path based method OBsp and a direct-overlap method OBoverlap, to reveal obesity-disease connections at protein-interaction subnetworks corresponding to thousands of biological functions and pathways. Through literature mining, we also curated an obesity-associated disease list, by which we compared the methods. As a result, OBNet outperforms other two methods. OBNet can predict whether a disease is obesity-related based on its associated genes. Meanwhile, OBNet identifies extensive connections between obesity genes and genes associated with a few diseases at various functional modules and pathways. Using breast cancer and Type 2 diabetes as two examples, OBNet identifies meaningful genes that may play key roles in connecting obesity and the two diseases. For example, *TGFB1* and *VEGFA* are inferred to be the top two key genes mediating obesity-breast cancer connection in modules associated with brain development. Finally, the top modules identified by OBNet in breast cancer significantly overlap with modules identified from TCGA breast cancer gene expression study, revealing the power of OBNet in identifying biological processes involved in the disease.

## INTRODUCTION

Obesity is a medical condition of accumulating too much body fat, which may have serious negative effects on human health. For example, it is known that obesity plays an important role in the development of many diseases, such as Type 2 diabetes (T2D), hypertension, cardiovascular disease, coronary heart disease and several types of cancers [1–5]. Obesity can also cause chronic low-grade inflammation, which contributes to the occurrence and development of metabolic disorders [6, 7]. In the past twenty years, there is a sustained increase in obese population, and the levels of overweight persons in many countries have already reached epidemic proportions [8], which presents an urgent need to study obesity and its association with multiple obesity-related diseases (ORDs).

At present, most studies focus on single mechanisms in connecting obesity and multiple ORDs. For example, Ersilia et al. studied the role of adiponectin in obesity and obesity-related diseases and find that expression enhancement of adiponectin may represent a useful therapeutic method against obesity and ORDs [9]. There are also studies targeting the association between obesity and specific diseases. For example, Zhang et al. used an integrated network of obesity and T2D to study their connections [10]. However, a global view of the association between obesity and ORDs simultaneously incorporating most biological processes and pathways, and across multiple disease types is more or less missing. The key bottleneck is the lack of sufficient knowledge on the diseases at the whole genome and transcriptome levels.

Recently, with the rapid advances of various sequencing techniques, we are posed at a better position to fix this gap. First, our knowledge about obesity and disease associated genes has been expanded quickly. For example, the genome-wide association study (GWAS) Catalog provides a quality controlled, manually curated and literature-derived collection of all published association studies, which has assessed more than 100,000 SNPs and their potential association with different traits and diseases [11]. By incorporating the SNP and gene associations, one can infer trait/disease associated genes through the GWAS Catalog [12]. The online Mendelian inheritance in man (OMIM) database also curates a comprehensive and authoritative compendium of human gene and known Mendelian disorders. By now, it has collected the information on all known Mendelian disorders and over 15,000 genes [46]. In addition, differential gene expression analysis and regression analysis between the control and disease samples provide data-driven approaches to infer tissue-specific obesity and disease associated genes [13, 14].

Second, our knowledge about gene interactions and gene signalling cascades has been enriched. For example, there are various protein-protein interaction databases including human protein reference database (HPRD) [15], STRING [16], and so on. In addition, the tissue and disease specific gene co-expression and regulatory networks could be inferred from databases like genotype-tissue expression (GTEx) [17] and the cancer genome atlas (TCGA) [18] using machine learning based methods like the weighted gene co-expression network analysis (WGCNA) [19] and the Bayesian network. As a result, it is possible to study the interaction between various types of gene signatures on multiple-level molecular networks. For example, Wang et al. constructed a network approach to analyse the connections among aging and a few diseases [20]. Guney et al. proposed a network-based in silico drug screening using the shortest path between drug targets and disease genes in a protein interaction network [21].

In this paper, we have developed and compared three different algorithms to identify the putative connections between obesity and obesity-related disease, namely (1) a random walk and gene set enrichment based network method called OBNet, (2) a shortest path based network method called OBsp and (3) a direct gene set overlap based method call OBoverlap. Using these methods, we try to answer a few questions including: (1) Which diseases are more relevant to obesity at molecular level? (2) Is there any common biological function or pathway involving in the connection between obesity and many ORDs? (3) Is there any disease specific obesity related biological function or pathways? (4) What genes are critical in mediating the connection between obesity and ORDs?

## RESULTS

Previous studies suggested that the network concept can explain the connections between many traits and diseases [12, 20, 21]. As such, we modelled the obesity-disease association by the mutual reachability between obesity and ORD genes in protein interaction networks. Here the concept of reachability was employed to describe potential interaction (or mutual influence) between obesity and disease genes. Generally speaking, if two genes are close in a network, they might interact with each other. We developed 3 methods called OBNet, OBsp, and OBoverlap to quantify this reachability. Since the obesity-disease association might be enriched in a few biological processes [9], we also studied the network reachability on specific biological processes or pathways.

### An overview of OBNet, OBsp and OBoverlap

**Figure 1:**
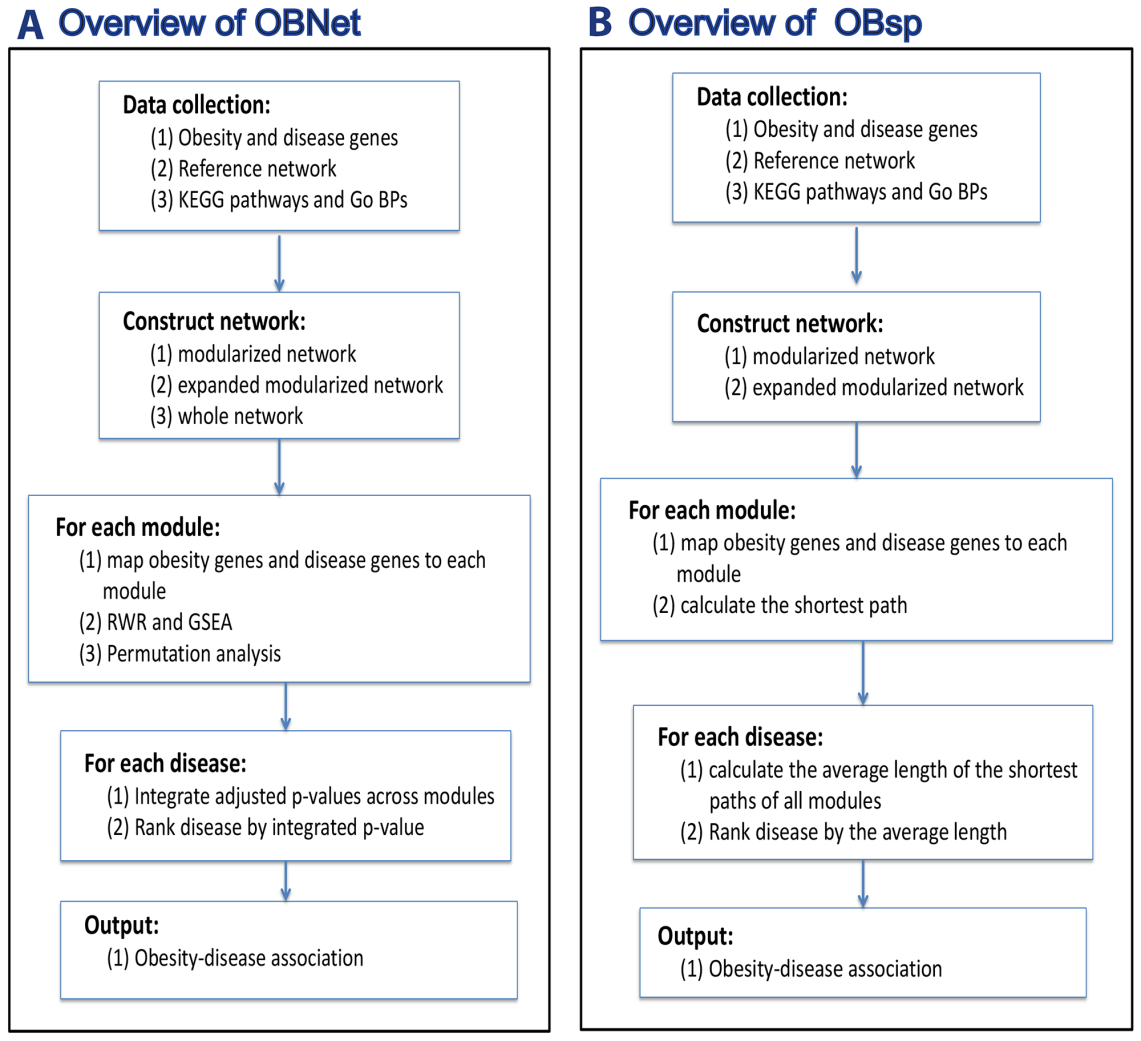
An overview of **(A)** OBNet and **(B)** OBsp. OBNet: A list of obesity and disease genes, a reference network, GO biological processes and KEGG pathways are first collected. The genes in specific GO term and KEGG pathway are mapped onto the reference network to define a modularized network, which could be further expanded by a random walk with restart (RWR) procedure to construct an expanded modularized network. After that, the obesity genes and disease genes are mapped to each (expanded) modularized network. The mutual reachability between obesity genes and disease genes is estimated by using RWR and a gene set enrichment analysis (GSEA). The significance of the mutual reachability is evaluated by using a permutation analysis, in which the obesity genes are randomly permuted, and the significance p-value is adjusted for multiple testing. Finally, the diseases are ranked by the minimum adjusted p-values across all (expanded) modularized networks and those with low adjusted p-values are obesity-related. OBsp: The mutual reachability of obesity and disease genes is estimated by the average shortest path between the two sets.

We presented an overview of the OBNet and OBsp algorithms in Figure 1. The major steps of OBNet were shown in Figure 1A, which is similar to our previous algorithm GeroNet [12]. Specifically, a list of obesity and disease genes, a reference network, GO biological processes and KEGG pathways were first collected. The genes in specific GO term and KEGG pathway were mapped onto the reference network to define a modularized network, which could be further expanded by a random walk with restart (RWR) procedure to construct an expanded modularized network. The (expanded) modularized networks present a network view of specific functions related to the GO function or KEGG pathway that defines it. After that, the obesity genes and disease genes were mapped to each (expanded) modularized network. The mutual reachability between obesity genes and disease genes was estimated by using RWR and a gene set enrichment analysis (GSEA) on three types of networks including modularized network, expanded modularized network and the whole network, corresponding to OBNet-Modularized network, OBNet-Expanded modularized network, and OBNet-Whole network respectively. The significance of the mutual reachability was evaluated by using a permutation analysis, in which the obesity genes are randomly permuted, and the significance p-value is adjusted for multiple testing. Finally, the diseases were ranked by the minimum adjusted p-values across all (expanded) modularized networks and those with low adjusted p-values are obesity-related. By this way, we also identified GO biological processes and KEGG pathways in which obesity and disease genes are significantly associated (or reachable). The details of each step are presented in Materials and Methods.

OBsp (OBsp-Modularized network and OBsp-Expanded modularized network) is generally similar to OBNet (OBNet-Modularized network and OBNet-Expanded modularized network). The difference is that OBsp evaluates the reachability of obesity and disease genes by their shortest path in the (expanded) modularized network (see Figure 1B and Materials and Methods). OBoverlap calculates the Jaccard coefficient concerning obesity genes and disease genes and ranks the diseases based on this coefficient.

### Comparison of OBNet, OBsp and OBoverlap

We adopted the obesity genes and disease genes from our previous work [12], which merges GWAS cataolg and OMIM disease genes (Supplementary Dataset 1), and used STRING PPI Network with confidence level 400 as the reference network. We then compared the 3 methods by its accuracy in predicting obesity-related diseases. Towards this purpose, we constructed a gold set of obesity-related diseases based on literature mining, which consists of 51 diseases (Supplementary Table 1). Specifically, we first searched all PubMed abstracts between 2009 and 2015, and ranked the diseases according to the Jaccard coefficient between abstracts in which the disease name and the term “obesity” occur (Supplementary Dataset 2). To make sure that the 51 diseases are really ORDs, we also searched literatures to confirm their association to obesity (Supplementary Dataset 3).

**Figure 2:**
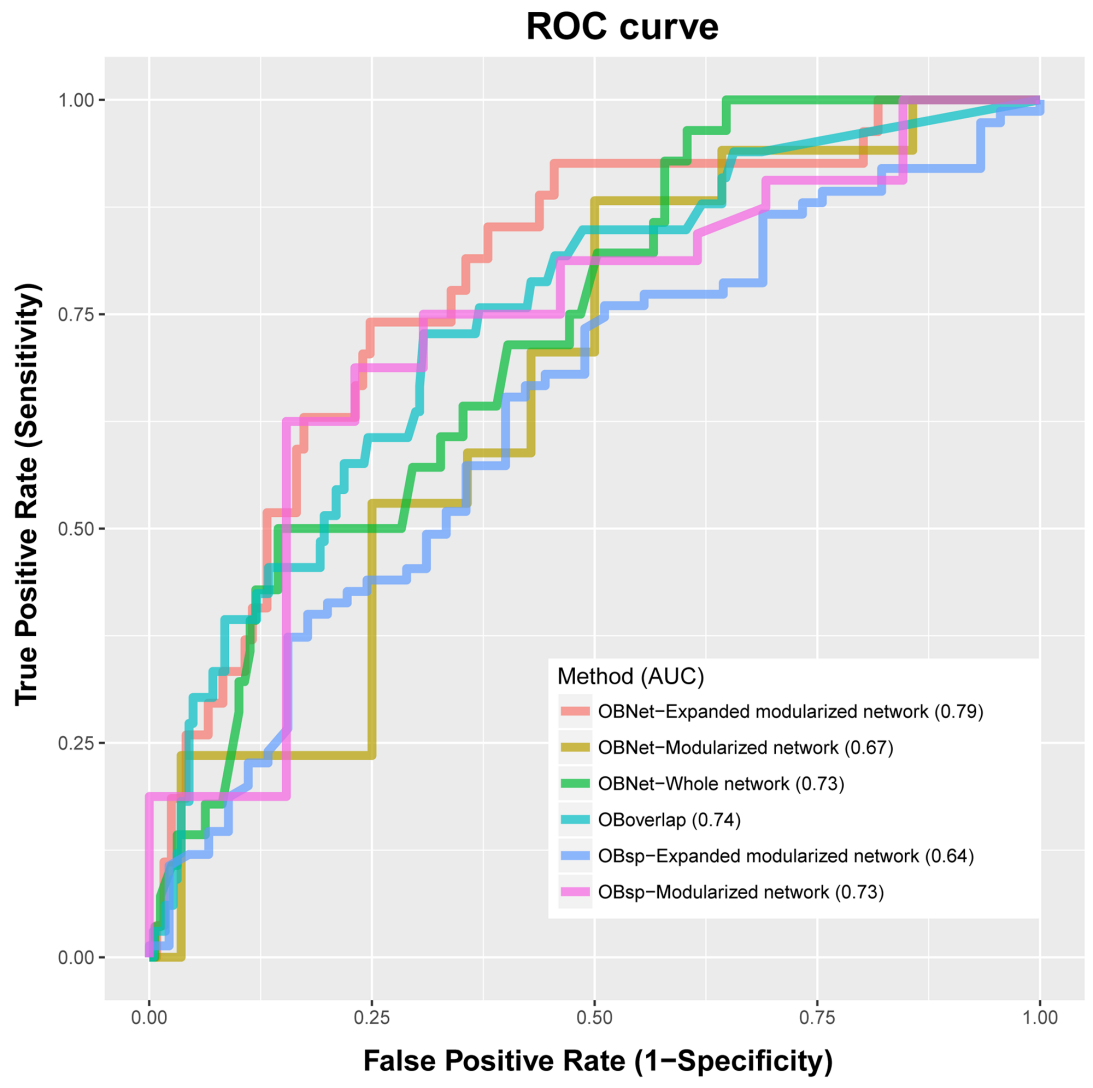
Comparison of OBNet, OBsp and OBoverlap OBNet-Expanded modularized network represents OBNet using expanded modularized network; OBNet-modularized network represent OBNet using modularized network; other methods are defined similarly.

We compared 6 methods, i.e., OBNet-Modularized network, OBNet-Expanded modularized network, OBNet-Whole network, OBsp-Modularized network, OBsp-Expanded modularized network and OBoverlap, in predicting ORDs in the gold set and plotted their receiver operating characteristic curves (ROCs) in Figure 2 and sensitivity in Supplementary Figure 2. OBNet-Expanded modularized network has an area under curve (AUC) 0.79, outperforming other methods. It indicates that the association between obesity and ORDs are possibly mediated by a few biological processes and pathways, and different pathways may contribute differently to the association [9]. It is interesting that direct overlapping performs better than shortest path based methods, which are commonly used in studying the connections between traits like drugs and diseases [21]. As a suggestion, the selection of appropriate computational model is critical in data-driven studies. We adopted the best model OBNet-Expanded modularized network in all following studies.

### Obesity related diseases predicted by OBNet-Expanded modularized network

We listed in Table 1 the top 40 diseases based on its association with obesity, and presented a full table of 147 diseases in Supplementary Table 2. As a criterion to evaluate obesity, body mass index ranks first, which is followed by autism spectrum disorder-bipolar disorder-schizophrenia. It has been well known that people especially children with autism spectrum disorder have a prevalence of obesity [22]. Interestingly, parental obesity is also a risk factor for children autism spectrum disorder [23], which indicates that autism and obesity are truly interacting with each other. However, the mechanisms behind the interaction is rarely known, on which the functional modules mediating the interaction might shed some lights.

**Table 1:** Top 40 predicted obesity associated diseases

Disease	FDR	Disease	FDR
Body mass index	3.15E-32	Crohn’s disease	7.96E-07
Autism spectrum disorder-bipolar disorder-schizophrenia	7.60E-11	Asthma	8.48E-07
Coronary artery disease	3.29E-09	Red blood cell traits	1.05E-06
Type 2 diabetes	3.47E-09	Prostate cancer	1.16E-06
Metabolite levels	7.60E-09	Fasting plasma glucose	1.68E-06
Atrial fibrillation	8.76E-09	Celiac disease	1.70E-06
Height	9.42E-09	Blood pressure	2.25E-06
Obesity-related traits	5.26E-08	Rheumatoid arthritis	2.50E-06
Chronic lymphocytic leukemia	5.51E-08	Inflammatory biomarkers	4.07E-06
Type 1 diabetes	6.71E-08	HDL Cholesterol - Triglycerides (HDLC-TG)	4.73E-06
Bone mineral density	9.23E-08	Lipid traits	1.25E-05
Blood trace element (Cu levels)	1.34E-07	Platelet counts	1.85E-05
Neuroblastoma	1.34E-07	Allergic sensitization	1.90E-05
Warfarin maintenance dose	1.34E-07	Pulmonary function	1.94E-05
C-reactive protein	1.36E-07	Breast cancer	2.03E-05
Metabolic syndrome	1.47E-07	Electrocardiographic traits	2.38E-05
HDL cholesterol	1.77E-07	Inflammatory bowel disease	2.87E-05
Alzheimer’s disease	2.21E-07	Schizophrenia or bipolar disorder	2.88E-05
Thyroid function	2.24E-07	Immune response to smallpox vaccine (IL-6)	3.34E-05
Triglycerides	6.69E-07	Fibrinogen	3.46E-05

Further navigating the list, we find a few heart diseases such as coronary artery disease and atrial fibrillation, metabolic diseases and traits such as Type 2 diabetes and HDL cholesterol, and cancers such as chronic lymphocytic leukaemia and Breast cancer. The connection between heart diseases and obesity has been well recognized. According to American Heart Association, obese can raise blood cholesterol, which increases blood pressure and induces many heart diseases like coronary artery disease (http://www.heart.org/HEARTORG/HealthyLiving/WeightManagement/Obes-ity/Obesity-Information_UCM_307908_Article.jsp#.WO5TYPnyvcs). Many studies have shown that obesity is associated with diabetes especially Type 2 diabetes [24, 25]. For example, a cross-sectional study revealed that 75% of the patients with Type 2 diabetes in Brazil are overweight (BMI>25 kg/m^2^), among which 30% are obese [26]. The proportions of overweight Type 2 diabetes patients are 85% and 86% respectively in the United Kingdom [27] and United States (Centers for Disease Control and Prevention (CDC) 2004) and those for obesity are 52% and 55% respectively for the two countries. Similarly, of patients with Type 1 diabetes, 55.3% are overweight (BMI ≥25 kg/m^2^), 16.6% are obese (BMI ≥30 kg/m^2^), and 0.4% have morbid obesity (BMI ≥40 kg/m^2^). Finally, it is of note that the association between obesity and a few cancers has also brought wide concerns (https://well.blogs.nytimes.com/2016/08/24/obesity-linked-to-at-least-13-types-of-cancer/?_r=0).

According to breast cancer research in Unite Kingdom, scientists estimated that 7% to15% breast cancer cases are caused by obesity in developed countries [28–30] and two cohort studies based on Cancer Research UK study and the Million Women Study have found that obese women have a 30% higher risk of postmenopausal breast cancer than women with a healthy weight [31, 32].

It is worth to note that several diseases in Table 1were not classified as ORD in the gold ORD set (Supplementary Table 1), e.g., metabolic syndrome, thyroid function and HDL cholesterol. They are possible false-positive predictions by OBNet. However, we did find evidences to support their connection with obesity. For example, Rashild and Genest found that obesity can increase HDL cholesterol, which is associated with the development of coronary artery disease [33]. More attention should be paid to these diseases.

### Function modules mediating obesity and diseases

**Figure 3:**
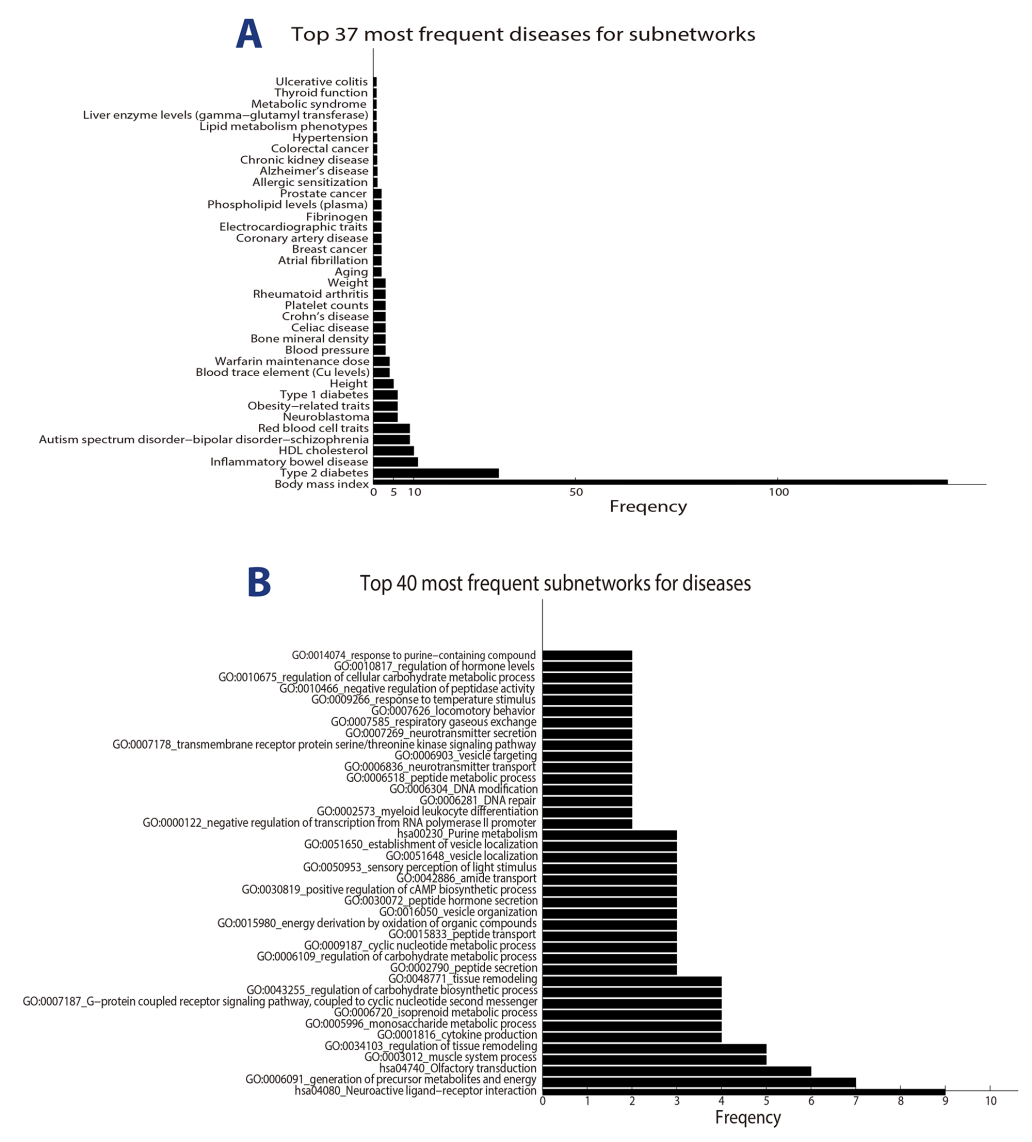
The connections between obesity and ORDs **(A)** Top 37 most frequent obesity-related diseases for OBNet based on expanded modularized network. Here frequency means the number of subnetworks in which obesity and the disease are significant connected. **(B)** Top 40 most frequent significant modules for ORDs. Here frequency means the number of diseases significantly connected with obesity in the module.

Since the function modules mediating obesity and ORDs might be critical in revealing the biology underlying their connections, we zoomed into the significant modules, in which obesity genes and disease are significantly interacted (at FDR 0.05) for the GSEA analysis. As a result, we identified 1232 disease-module pairs involving 781 unique functional modules (Supplementary Dataset 4). Clearly, a disease should be more obesity-related if it interacts with obesity in multiple function modules. We plotted in Figure 3A the top 37 ORDs accordingly to their number of significant function modules. Body mass index, Type 2 diabetes, and inflammatory bowel disease (IBD) ranks in top 3. The interaction of obesity and IBD is a hot topic in recent years due to their highly prevalent in western societies. For example, Flores et al. showed that obesity is highly prevalent in IBD patients in the US population [34]. A recent study suggested that the association may related to share dietary or environmental exposures that exert their effect through changes in the intestinal microbiota [35].

It is of note that different function modules play different roles in mediating obesity-disease association. Some modules (networks) mediate the connection of obesity and a wide range of diseases, while others are disease specific. We plotted in Figure 3B the top 40 networks most frequently involved in obesity-disease interactions. Metabolic associated modules are most prevalent in the figure. For example, GO:0006091_generation of precursor metabolites and energy, GO:0005996_monosaccharide metabolic process and GO:0006720_isoprenoid metabolic process rank at top 2, 7 and 8 respectively. It is known that obesity has a significant impact on the macronutrient metabolisms, which might be a key factor to induce obesity related diseases [36].

### Network view of the connection between obesity and ORDs and the key connector genes

**Figure 4:**
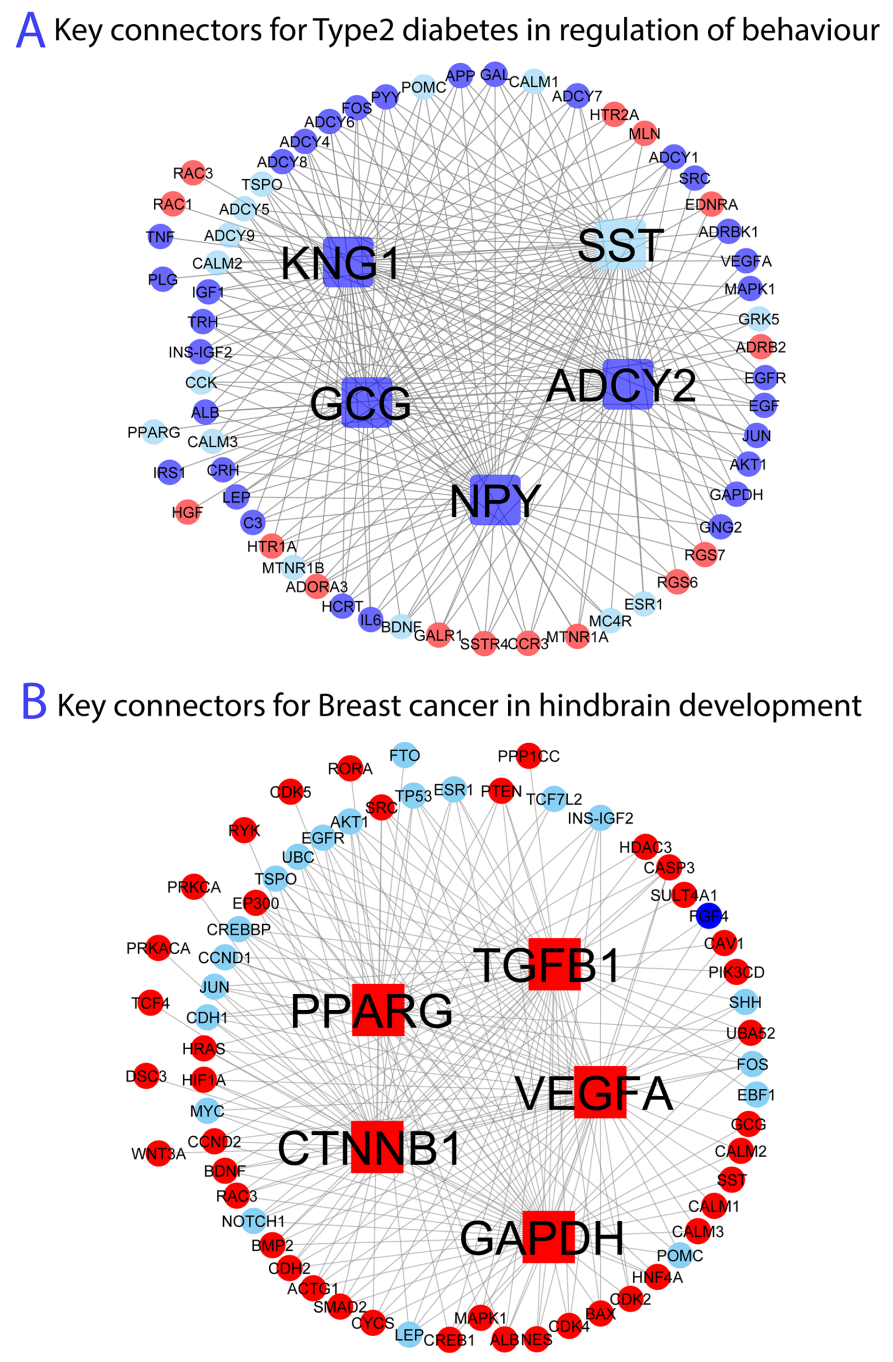
Network topology and key genes connecting **(A)** obesity and Type 2 diabetes in regulation of behaviour and **(B)** obesity and breast cancer in hindbrain development. We use node shape to denote key connectors: (1) square represents the top 5 key connectors; (2) circle represents expanded obesity and disease genes. We use fill colour to denote new (expanded) obesity and disease information: (1) red represents obesity gene; (2) blue represents disease gene.

For a better view of the network modules in mediating obesity-disease associations, we plotted obesity genes and diseases genes in the significant subnetworks. In addition, we performed key connector analysis (KCA, see Materials and Methods for details) to infer key genes connecting obesity and ORDs [12, 55]. KCA has been proven to be effective in identifying important genes associated with a set of target genes in a network [10, 12]. We selected two commonly believed obesity related diseases including Type 2 diabetes and breast cancer as case studies to illustrate the connection network and key connectors (Figure 4).

#### Case study 1: Type 2 diabetes

The connection between Type 2 diabetes and obesity is most significant on the module corresponding to GO:0050795_regulation of behaviour with FDR 1.71E-6 (Supplementary Dataset 4). Thus, we focused on the subnetwork associated with the function “regulation of behavior”, which consists of 497 genes and 23,135 interactions. We then performed KCA of the connecting genes on the subnetwork. For a better view, we retrieved the subnetwork consisting of the top 5 key connector genes and their neighbouring genes (see Figure 4A). As we can see, top 5 key connector genes are *ADCY2*, *NPY*, *GCG*, *KNG1* and *SST*, respectively, among which *ADCY2* is most significant. Adenylyl cyclase type 2 (*ADCY2*) encodes a member of the family of adenylyl cyclases, which are membrane-associated enzymes that catalyze the formation of the secondary messenger cyclic adenosine monophosphate (cAMP) from ATP. Interestingly this gene has protein interactions with many known obesity genes (e.g., *MC4R, POMC, ADRB2, ADCY9 and BDNF*) and also many known Type 2 diabetes associated genes (*INS-IGF2, GRK5, ADCY5*) within the subnetwork, supporting its critical role in mediating the Type 2 diabetes-obesity interaction in the function module. In addition, other top key connections such as *NPY* and *GCG* are also related to both obesity and Type 2 diabetes in humans [37].

#### Case study 2: Breast cancer

The connection between breast cancer and obesity is most significant on the module corresponding to GO:0030902_hindbrain development with FDR 1.74E-3 (Supplementary Dataset 4). We focused on the subnetwork corresponding to the function “hindbrain development”, which consists of 500 genes and 8186 interactions. Similarly, we performed KCA of the connecting genes and retrieved the subnetwork consisting of the top 5 key connector genes and their neighbouring genes (see Figure 4A). As can be seen from Figure 4A, the top 5 key connector genes are *TGFB1*, *VEGFA, CTNNB1, GAPDH* and *PPARG*, respectively, among which *TGFB1* is most significant. Transforming Growth Factor Beta 1 (*TGFB1*) secrets protein that performs many cellular functions, including the control of cell growth, cell proliferation, cell differentiation and apoptosis [38]. A few studies have suggested that *TGFB*1 is critical to both obesity and breast cancer. For example, Yadav et al. suggested that *TGFB1/SMAD3*-regulated white adipose tissue (WAT) transcriptome in a mouse model of diet induced obesity; and Candida et al. has shown a mechanistic relationship between *TGFB1* and breast cancer [39]. As a result, TGFB1 might play roles in connecting obesity and breast cancer. Other top key drivers like *VEGFA*, *GAPDH* and *PPARG* also play a role in breast cancer and obesity [12, 40].

### Validation of OBNet by a gene expression study

In addition to the numerous literature supported findings identified by OBNet, we also considered an orthogonal validation by using gene expression data. Specifically, we downloaded the gene expression data of 531 breast cancer 62 matched normal samples from The Cancer Genome Atlas (TCGA, http://cancergenome.nih.gov/) (on Dec 16, 2015). We then applied WGCNA to construct gene co-expression modules from the 531 cancer samples, and achieved 29 co-expression modules. After that, we performed module differential connectivity (MDC) [41] to identify the modules significantly perturbed by breast cancer. Specifically, for each module, MDC calculates the ratio between the average connectivity of all gene pairs for breast cancer samples and that of gene pairs for normal samples. A module with MDC larger than (less than) 1 gains (loses) connectivity when changing from normal to cancer state. The significance of MDC is estimated by a permutation study on the samples [13, 41]. As such, there are 10 modules significantly perturbed (at FDR 0.05) by breast cancer (see Supplementary Table 3 and Supplementary Dataset 5).

We then compared the 10 MDC modules with the top 10 modules mediating the interaction between breast cancer and obesity as identified by OBNet (Supplementary Table 4). Specifically for each module pair, we performed a Fisher’s exact test on the overlap of their genes and calculated adjusted p-values using Benjamini Hochberg method. The adjusted p-values are shown in Table 2. As can be seen, 6 of OBNet modules (i.e., GO:0033135_regulation of peptidyl-serine, GO:0030003_cellular cation homeostasis, GO:0006521_regulation of cellular amino acid metabolic process, GO:0048871_multicellular organismal homeostasis, GO:0055080_cation homeostasis, GO:0055082_cellular chemical homeostasis) are significantly overlapped with at least one module from MDC (FDR<0.05). At the same time, 3 of MDC modules are also significantly overlapped with OBNet modules. We then performed a permutation study to assess the significance of the overlap (between the top 10 modules of OBNet and MDC). We randomly shuffled the genes in the 29 WGCNA modules (keeping the numbers of genes in each module), selected the top 10 differential modules by MDC, and overlapped the modules with OBNet modules. The process was repeated for 1000 times. We then calculated number of significant overlapped OBNet modules for each run (see Supplementary Figure 1). As a result, the mean number of significant overlap is 0.67 (±1.22) and the p-value of observed 6 or more significant overlaps is 5.16E-6 by assuming a normal distribution. As an indication, our method could help to find some significant co-expression modules associated with breast cancer without considering gene expression data.

**Table 2:** Comparing the top 10 modules associated with Breast cancer identified by WGCNA and by our study

module	blue	darkgreen	darkorange	darkgrey	royalblue	lightgreen	orange	white	saddlebrown	skyblue
GO:0030902_hindbrain development	1	1	1	1	1	1	1	1	1	1
GO:0033135_regulation of peptidyl-serine phosphorylation	9.78E-09	3.90E-02	1	1	1	1	1	1	1	1
GO:0021695_cerebellar cortex development	1	1	1	1	1	1	1	1	1	1
GO:0030003_cellular cation homeostasis	1.61E-11	1	1	1	1	1	1	1	1	1
GO:0022037_metencephalon development	1	1	1	1	1	1	1	1	1	1
GO:0006521_regulation of cellular amino acid metabolic process	1	3.88E-02	8.78E-02	4.04E-01	9.78E-09	1	1	1	1	1
GO:0048871_multicellular organismal homeostasis	7.17E-08	1	1	1	1	1	1	1	1	1
GO:0055080_cation homeostasis	3.96E-09	1	1	1	1	1	1	1	1	1
GO:0055082_cellular chemical homeostasis	2.00E-10	1	1	1	1	1	1	1	1	1
GO:0031145_anaphase-promoting complex-dependent proteasomal ubiquitin-dependent protein catabolic process	1	1.30E-01	2.52E-01	2.89E-01	8.53E-01	1	1	1	1	1

The co-expression modules were named by colors and the genes in each module were listed in Supplementary Dataset 5.

## DISCUSSION

In this paper, we used three different methods to identify the connections between obesity and obesity-related diseases, namely OBNet, OBsp and OBoverlap. OBNet on expanded modularized network outperformed other methods, indicating that the interaction between obesity genes and ORD genes might enriched in specific functional modules and pathways. The observation is supported by many literatures. For example, Gluckman and Hanson found that developmental and epigenetic pathways are critical in connecting obesity and diseases [42]. Singla et al. identified the roles of metabolic functions and pathways in connecting obesity and metabolic diseases [36].

It is worth noting that GWAS and OMIM disease genes might suffer from false-positives and incompleteness, and thus the obesity and disease signatures used in this study might not be very accurate. Nevertheless, OBNet can predict obesity associated diseases based on these genes with reasonable accuracy. With the accumulation of our knowledge of diseases and obesity, the performance of OBNet could be further improved in the future. In addition, OBNet can identify important disease associated modules from gene expression study, which also confirm its potential in disease studies.

However, OBNet has a few limitations. First of all, OBNet does not reflect any tissue specificity. It is known that at different tissues, obesity might correlate with different diseases. For example, adipose tissue dysfunction relates obesity to diabetes and vascular diseases [43]. A possible solution is to make use of tissue-specific networks constructed from tissue specific data such as Genotype-Tissue Expression (GTEx) [17]. Unlike PPI network which reflect general protein interactions, this kind of network can catch more tissue specific gene interactions. Second, the mutual reachability of genes alone might not reflect all aspect of gene interactions. In OBNet, we treat each gene with equal importance, which is not generally true. A future direction is to infer the importance of obesity and disease genes based on their roles in shaping obesity and diseases, and combine the information into the algorithm. Third, it is known that GO and KEGG have some overlap, which might have some influences to the algorithms. However, a previous study suggests that the influence might not be critical [12]. Finally, it might be useful to integrate various omics data like gene expression into OBNet.

Finally, though we studied the interaction between obesity and diseases in this study, the three methods proposed may have some further applications. In principle, they could be used to study the interactions between any two traits and diseases. For example, by studying the reachability of drug target or perturbed genes and disease genes, one can predict the sensitivity of a drug to a specific disease and meanwhile infer the major biological functions and pathways involved in drug response. Another interesting topic is to study the interaction between environmental factors (like smoking, drinking or microbes) and diseases. However, it is out of the scope of this study.

## MATERIALS AND METHODS

### Collection of data and data pre-processing

In this paper, the obesity genes and disease genes were obtained from NIH GWAS Catalog and Online Mendelian Inheritance in Man (OMIM) [44]. By merging the two studies, a list of genes about 257 diseases were obtained (see Supplementary Dataset 1). The detailed merging method was provided in our recent work (Yang et al. 2016b).

The reference protein-protein interaction (PPI) network was extracted from Search Tool for the Retrieval of Interacting Genes/Proteins (STRING) [45]. In this paper, we adopted STRING400 network, i.e., STRING PPI network with median (400) confidence. Finally, we downloaded 2968 GO BPs from Gene ontology databases and 197 KEGG pathways to generate various network modules.

### Constructing gold ORD set

We applied a literature based text-mining to evaluate whether a disease is associated with obesity. Specifically, we ranked a disease according to the Jaccard coefficient between the disease name and the term “obesity” in PubMed abstracts published from 2009 to 2015 (Supplementary Dataset 2). The PubMed abstracts containing the term “obesity” from 2009 to 2015 were retrieved by using Entrez Programming Utilities (http://eutils.ncbi.nlm.nih.gov/entrez/eutils/esearch.fcgi?db=pubmed&term=[obesity]+AND+2009:2015[pdat]&retmax=999999), and the abstracts containing disease name and both disease name and “obesity” were retrieved similarly. The Jaccard coefficient is calculated as a reasonable measure to represent the co-occurrence of obesity and a disease [46].

$$Jaccard\left(disease,obesity\right)=\left|\frac{PubMedID_{disease}\cap PubMedID_{obesity}}{PubMedID_{disease}\cup PubMedID_{obesity}}\right|$$ (1)

Where *PubMedID*_*disease*_ and *PubMedID*_*obesity*_ are the PubMed IDs corresponding to the PubMed abstracts containing the disease name and the term “obesity”, respectively.

Based on our knowledge, we selected diseases with Jaccard coefficient larger than or equal to 0.004 as obesity-related diseases. However, it is of note that there are some obvious obesity-related diseases without taking into account. According to previous study, there are some diseases associated with obesity, such as cardiovascular diseases, metabolic diseases, serious psychiatric illness [47], coronary heart disease [48], Alzheimer’s disease [49] and inflammatory diseases [50]. Therefore, schizophrenia, major depressive disorder, inflammatory bowel disease, Alzheimer’s disease and other four diseases are added to the list. At last, we obtained 51 diseases that are defined as ORDs, which are used for our study (Supplementary Table 1). To further validate the 51 diseases as ORDs, we also provided literatures to support them (Supplementary Dataset 3).

### Methods to identify obesity-disease association

We used three algorithms to identify the association between obesity and diseases, namely OBNet based on a procedure similar to gene set enrichment analysis and a random walk with restart procedure, OBsp performed by using the shortest path algorithm, and OBoverlap based on direct overlapping between obesity and disease associated genes.

#### OBNet

OBNet is generally similar to our previous software GeroNet [12]. Specifically, we first mapped different KEGG pathway genes or GO BP genes to the PPI network to generate a variety of modularized networks. It is worth to note that we only used GO BPs or KEGG pathways with the number genes less than 500 and ignored those with overly large gene sets. Based on each modularized network, an expanded modularized network was obtained by using a random walk with restart (RWR) procedure until it reaches 5 times the original gene size or a maximum of 500 genes. A modularized network or an expanded modularized network is considered as a module. Second, we mapped obesity genes and disease genes to each module or the whole PPI network and then use RWR and a procedure which is similar to gene set enrichment analysis (GSEA) to estimate the mutual reachability between obesity genes and disease genes (see below). If the number of obesity genes or disease genes mapped to network are too few, RWR may not do well. So we only consider modularized networks or expanded modularized networks that contain at least 5 obesity genes and 5 disease genes. Each of diseases is estimated respectively. After that, a permutation test is used to estimate the significance of the reachability between obesity genes and disease genes by randomly permute the obesity genes.

*RWR*: For a PPI network *G* = (*V*, *E*) which contains a set of proteins *V* and a set of interactions *E*, an *n* × *n* adjacency matrix *A* is used to represent the PPI network, where *n* is the number of proteins. The entry at row *i* and column *j* will be set to 1 if there is an interaction between protein *i* and protein *j*; otherwise it will be set to 0. The adjacency matrix *A* is then normalized as following

$$A_{\left[i,j\right]}^{\prime }=\frac{A_{\left[i,j\right]}}{\sum_{k=1}^{n}A_{\left[k,j\right]}}$$ (2)

The random walker algorithm starts from a set of seed genes, such as obesity, disease genes or modularized network genes. The initial state *P*_0_ is represented by a column vector $P_{0}={\left[\psi_{1}\text{,}\psi_{2}\text{,}\geq \text{,}\psi_{\text{n}}\right]}^{T}$, where *ψ*_*i*_ is set to $\frac{1}{\text{m}}$ for the *m* seed genes and 0 for other genes located on the network. It then randomly visit adjacent genes in every tick of time (t→t+1). The state probabilities *P*_*t*+1_ at time *t* + 1 is calculated as following

$$P_{t+1}=\left(1-r\right)A^{\prime }P_{t}+rP_{0}$$ (3)

where *P*_*t*_ is the probabilities at time *t*, *r* is the restart probability (i.e., starting again from the seed genes). For simplity, we set *r* to be 0.5 in this study. This process will be stopped if it reaches a steady-state when the difference between *P*_*t*_ and *P*_*t*+1_ is smaller than 1e-6 used by previous studies [51].

*Obesity-disease association on module:* We use a method similar to GSEA to calculate a score which is used to indicate the reachability between a set of obesity genes and a set of disease genes on module [52]. Using a set of disease genes as the starting points, we go across the sorted gene list of this module based on the probability of genes obtained by RWR, if we meet a gene not an obesity gene, $-\sqrt{\frac{G}{N-G}}$ is then added to the score, where *N* is the number of genes in the module, and *G* is the number of obesity genes; otherwise, $\sqrt{\frac{N-G}{G}}$ is added. This generates a curve and the peak value of the curve is defined as *ES*_1_. Similarly, we calculate *ES*_2_ using obesity genes as seed genes. The enrichment score is then defined as following

$$ES_{\beta }=\beta ES_{1}+\left(1-\beta \right)ES_{2}\;\text{with}\;0<\beta <1$$ (4)

In order to assess the significance level of *ES*_*β*_, we permute obesity genes in the module for 100 times to obtain the null distribution of enrichment scores. According to this, *ES*_*β*_ is converted to a normal z-score statistic and then a p-value is calculated and adjusted. After adjusting p-value of obesity-disease connection for multiple testing, we defined the p-value of obesity-disease association to be the minimum p-value for all relevant modules. The diseases are ranked based on their p-values.

We evaluated OBNet based on area under the curve (AUC) of receiver operating characteristic (ROC) curve by comparing inferred rank of diseases and the gold ORD list. As such, we set *β* to be 0.1 since it achieves the best performance.

#### OBsp

OBsp is generally similar to OBNet except that we used the shortest path to calculate the reachability of obesity and disease associated genes. Specifically for a given disease and a given module, we first calculated the shortest pathway of all disease and obesity gene pairs on the module by get.shortest.paths{igraph} function in R. The disease-obesity distance for the module was calculated as the average length of the shortest paths (of all gene pairs). Finally, the disease-obesity reachability was calculated as the minimum distances for all modules, and the diseases were ranked based on the reachability.

#### OBoverlap

OBoverlap calculates the Jaccard coefficient concerning obesity genes and disease genes and ranked according to the Jaccard coefficient.

### Key connector analysis

We adopted a previously established software package key driver analysis (KDA) [53] to identify key connectors in PPI network. KDA was originally designed to identify “key regulators” in a directed regulatory network. When applied to undirected networks like PPI networks, we consider the key nodes as “key connectors” since they do not necessarily contain the directional information [53]. Such key connectors function more like a “hub” gene, instead of being considered as “master regulators”. Specifically, KDA takes a set of genes G and an undirected gene network N as inputs. It has two searching strategies namely dynamic neighbourhood search (DNS) and static neighbourhood search (SNS) for identifying key connectors. We adopted DNS in this study: (1) It first generates a subnetwork *N*_*G*_ consisting of all nodes in N with no more than L (*L* = 2 in this study) steps away from the nodes in G. (2) For each gene g in *N*_*G*_ DNS then searches for genes with distances no more than *h* = 1, 2, ≥, *H* (*H* = 2 in this study) in *N*_*G*_. The set of genes (not including g) is denoted by *N*_*G*_(*HLN*_*g*,*h*)_. The Hypergeometric test is then used to calculate the enrichment between *N*_*G*_(*HLN*_*g*,*h*)_ and G with the genes in *N*_*G*_ as background for each h. The final enrichment p-value of each gene g is calculated as the minimum p-value across h layers. (3) The Bonferroni correction is performed to adjust for multiple testing and the genes with significant Bonferroni p-values (≤ 0.05) are outputted as key connectors.

### Function enrichment

The function enrichment was done by David Bioinformatics Resources 6.8.

## SUPPLEMENTARY MATERIALS FIGURES,TABLES AND DATASETS















## References

[1] Bluher M (2013). Adipose tissue dysfunction contributes to obesity related metabolic diseases. Best Pract Res Clin Endocrinol Metab.

[2] Ohashi K, Shibata R, Murohara T, Ouchi N (2014). Role of anti-inflammatory adipokines in obesity-related diseases. Trends Endocrinol Metab.

[3] Herouvi D, Karanasios E, Karayianni C, Karavanaki K (2013). Cardiovascular disease in childhood: the role of obesity. Eur J Pediatr.

[4] Feola A, Ricci S, Kouidhi S, Rizzo A, Penon A, Formisano P, Giordano A, Di Carlo A, Di Domenico M (2017). Multifaceted breast cancer: the molecular connection with obesity. J Cell Physiol.

[5] Marengo A, Rosso C, Bugianesi E (2016). Liver cancer: connections with obesity, fatty liver, and cirrhosis. Annu Rev Med.

[6] Ouchi N, Parker JL, Lugus JJ, Walsh K (2011). Adipokines in inflammation and metabolic disease. Nat Rev Immunol.

[7] Donath MY, Shoelson SE (2011). Type 2 diabetes as an inflammatory disease. Nat Rev Immunol.

[8] Finucane MM, Stevens GA, Cowan MJ, Danaei G, Lin JK, Paciorek CJ, Singh GM, Gutierrez HR, Lu Y, Bahalim AN, Farzadfar F, Riley LM, Ezzati M (2011). National, regional, and global trends in body-mass index since 1980: systematic analysis of health examination surveys and epidemiological studies with 960 country-years and 9.1 million participants. Lancet.

[9] Nigro E, Scudiero O, Monaco ML, Palmieri A, Mazzarella G, Costagliola C, Bianco A, Daniele A (2014). New insight into adiponectin role in obesity and obesity-related diseases. Biomed Res Int.

[10] Zhang SY, Wang B, Shi JS, Li J (2015). Network-based association study of obesity and type 2 diabetes with gene expression profiles. Biomed Res Int.

[11] Welter D, MacArthur J, Morales J, Burdett T, Hall P, Junkins H, Klemm A, Flicek P, Manolio T, Hindorff L, Parkinson H (2014). The NHGRI GWAS Catalog, a curated resource of SNP-trait associations. Nucleic Acids Res.

[12] Yang JL, Huang T, Song WM, Petralia F, Mobbs CV, Zhang B, Zhao Y, Schadt EE, Zhu J, Tu ZD (2016). Discover the network mechanisms underlying the connections between aging and age-related diseases. Sci Rep.

[13] Liu XM, Yang JS, Zhang Y, Fang Y, Wang FY, Wang J, Zheng XQ, Yang JL (2016). A systematic study on drug-response associated genes using baseline gene expressions of the Cancer Cell Line Encyclopedia. Sci Rep.

[14] Yang JL, Huang T, Petralia F, Long Q, Zhang B, Argmann C, Zhao Y, Mobbs CV, Schadt EE, Zhu J, Tu ZD, GTEx Consortium (2016). Synchronized age-related gene expression changes across multiple tissues in human and the link to complex diseases (vol. 5, pg 15145, 2015). Sci Rep.

[15] Prasad TS, Goel R, Kandasamy K, Keerthikumar S, Kumar S, Mathivanan S, Telikicherla D, Raju R, Shafreen B, Venugopal A, Balakrishnan L, Marimuthu A, Banerjee S (2009). Human Protein Reference Database-2009 update. Nucleic Acids Research.

[16] Szklarczyk D, Franceschini A, Wyder S, Forslund K, Heller D, Huerta-Cepas J, Simonovic M, Roth A, Santos A, Tsafou KP, Kuhn M, Bork P, Jensen LJ (2015). STRING v10: protein-protein interaction networks, integrated over the tree of life. Nucleic Acids Res.

[17] Ardlie KG, DeLuca DS, Segre AV, Sullivan TJ, Young TR, Gelfand ET, Trowbridge CA, Maller JB, Tukiainen T, Lek M, Ward LD, Kheradpour P, Iriarte B (2015). The Genotype-Tissue Expression (GTEx) pilot analysis: multitissue gene regulation in humans. Science.

[18] Koboldt DC, Fulton RS, McLellan MD, Schmidt H, Kalicki-Veizer J, McMichael JF, Fulton LL, Dooling DJ, Ding L, Mardis ER, Wilson RK, Ally A, Balasundaram M (2012). Comprehensive molecular portraits of human breast tumours. Nature.

[19] Langfelder P, Horvath S (2008). WGCNA: an R package for weighted correlation network analysis. BMC Bioinformatics.

[20] Wang J, Zhang S, Wang Y, Chen L, Zhang XS (2009). Disease-aging network reveals significant roles of aging genes in connecting genetic diseases. PLoS Comput Biol.

[21] Guney E, Menche J, Vidal M, Barabasi AL (2016). Network-based in silico drug efficacy screening. Nat Commun.

[22] Curtin C, Jojic M, Bandini LG (2014). Obesity in children with autism spectrum disorder. Harv Rev Psychiatry.

[23] Suren P, Gunnes N, Roth C, Bresnahan M, Hornig M, Hirtz D, Lie KK, Lipkin WI, Magnus P, Reichborn-Kjennerud T, Schjolberg S, Susser E, Oyen AS (2014). Parental obesity and risk of autism spectrum disorder. Pediatrics.

[24] Weyer C, Funahashi T, Tanaka S, Hotta K, Matsuzawa Y, Pratley RE, Tataranni PA (2001). Hypoadiponectinemia in obesity and type 2 diabetes: close association with insulin resistance and hyperinsulinemia. J Clin Endocrinol Metab.

[25] Okada-Iwabu M, Yamauchi T, Iwabu M, Honma T, Hamagami K, Matsuda K, Yamaguchi M, Tanabe H, Kimura-Someya T, Shirouzu M, Ogata H, Tokuyama K, Ueki K (2013). A small-molecule AdipoR agonist for type 2 diabetes and short life in obesity. Nature.

[26] Gomes MB, Giannella Neto D, Mendonca E, Tambascia MA, Fonseca RM, Rea RR, Macedo G, Modesto Filho J, Schmid H, Bittencourt AV, Cavalcanti S, Rassi N, Faria M (2006). [Nationwide multicenter study on the prevalence of overweight and obesity in type 2 diabetes mellitus in the Brazilian population]. [Article in Portuguese]. Arq Bras Endocrinol Metabol.

[27] Daousi C, Casson IF, Gill GV, MacFarlane IA, Wilding JP, Pinkney JH (2006). Prevalence of obesity in type 2 diabetes in secondary care: association with cardiovascular risk factors. Postgrad Med J.

[28] Danaei G, Vander Hoorn S, Lopez AD, Murray CJ, Ezzati M, Collab CR (2005). Causes of cancer in the world: comparative risk assessment of nine behavioural and environmental risk factors. Lancet.

[29] Renehan AG, Soerjomataram I, Tyson M, Egger M, Zwahlen M, Coebergh JW, Buchan I (2010). Incident cancer burden attributable to excess body mass index in 30 European countries. Int J Cancer.

[30] Parkin DM, Boyd L (2011). Cancers attributable to overweight and obesity in the UK in 2010. Br J Cancer.

[31] Lahmann PH, Hoffmann K, Allen N, Van Gils CH, Khaw KT, Tehard B, Berrino F, Tjonneland A, Bigaard J, Olsen A, Overvad K, Clavel-Chapelon F, Nagel G (2004). Body size and breast cancer risk: Findings from the european prospective investigation into cancer and nutrition (EPIC). Int J Cancer.

[32] Reeves GK, Pirie K, Beral V, Green J, Spencer E, Bull D (2007). Cancer incidence and mortality in relation to body mass index in the Million Women Study: cohort study. BMJ.

[33] Rashid S, Genest J (2007). Effect of obesity on high-density lipoprotein metabolism. Obesity.

[34] Flores A, Burstein E, Cipher DJ, Feagins LA (2015). Obesity in inflammatory bowel disease: a marker of less severe disease. Dig Dis Sci.

[35] Harper JW, Zisman TL (2016). Interaction of obesity and inflammatory bowel disease. World J Gastroenterol.

[36] Singla P, Bardoloi A, Parkash AA (2010). Metabolic effects of obesity: A review. World J Diabetes.

[37] Rao AA, Sridhar GR, Srinivas B, Das UN (2008). Bioinformatics analysis of functional protein sequences reveals a role for brain-derived neurotrophic factor in obesity and type 2 diabetes mellitus. Med Hypotheses.

[38] Ghadami M, Makita Y, Yoshida K, Nishimura G, Fukushima Y, Wakui K, Ikegawa S, Yamada K, Kondo S, Niikawa N, Tomita H (2000). Genetic mapping of the Camurati-Engelmann disease locus to chromosome 19q13.1-q13.3. Am J Hum Genet.

[39] Perera CN, Chin HG, Duru N, Camarillo IG (2008). Leptin-regulated gene expression in MCF-7 breast cancer cells: mechanistic insights into leptin-regulated mammary tumor growth and progression. J Endocrinol.

[40] Leisner TM, Moran C, Holly SP, Parise LV (2013). CIB1 prevents nuclear GAPDH accumulation and non-apoptotic tumor cell death via AKT and ERK signaling. Oncogene.

[41] Zhang B, Gaiteri C, Bodea LG, Wang Z, McElwee J, Podtelezhnikov AA, Zhang C, Xie T, Tran L, Dobrin R, Fluder E, Clurman B, Melquist S (2013). Integrated systems approach identifies genetic nodes and networks in late-onset Alzheimer’s disease. Cell.

[42] Gluckman PD, Hanson MA (2008). Developmental and epigenetic pathways to obesity: an evolutionary-developmental perspective. Int J Obes (Lond).

[43] Hajer GR, van Haeften TW, Visseren FL (2008). Adipose tissue dysfunction in obesity, diabetes, and vascular diseases. Eur Heart J.

[44] Hamosh A, Scott AF, Amberger JS, Bocchini CA, McKusick VA (2005). Online Mendelian Inheritance in Man (OMIM), a knowledgebase of human genes and genetic disorders. Nucleic Acids Res.

[45] Franceschini A, Szklarczyk D, Frankild S, Kuhn M, Simonovic M, Roth A, Lin J, Minguez P, Bork P, von Mering C, Jensen LJ (2013). STRING v9.1: protein-protein interaction networks, with increased coverage and integration. Nucleic Acids Res.

[46] Levandowsky M, Winter D (1971). Distance between Sets. Nature.

[47] Walley AJ, Blakemore AI, Froguel P (2006). Genetics of obesity and the prediction of risk for health. Hum Mol Genet.

[48] Maffeis C, Tato L (2001). Long-term effects of childhood obesity on morbidity and mortality. Horm Res.

[49] Yamauchi T, Kadowaki T (2013). Adiponectin receptor as a key player in healthy longevity and obesity-related diseases. Cell Metab.

[50] Balistreri CR, Caruso C, Candore G (2010). The role of adipose tissue and adipokines in obesity-related inflammatory diseases. Mediators Inflamm.

[51] Hofree M, Shen JP, Carter H, Gross A, Ideker T (2013). Network-based stratification of tumor mutations. Nat Methods.

[52] Mootha VK, Lindgren CM, Eriksson KF, Subramanian A, Sihag S, Lehar J, Puigserver P, Carlsson E, Ridderstrale M, Laurila E, Houstis N, Daly MJ, Patterson N (2003). PGC-1alpha-responsive genes involved in oxidative phosphorylation are coordinately downregulated in human diabetes. Nat Genet.

[53] Zhang B, Zhu J

