# Supplementary material for: Using molecular functional networks to manifest connections between obesity and obesity-related diseases
Source: Oncotarget. 2017 Jul 22;8(49):85136–49. doi: 10.18632/oncotarget.19490 (PMC5689599; doi:10.18632/oncotarget.19490)
Supplement: Supplementary file 1 [file oncotarget-08-85136-s001.pdf]

Using molecular functional networks to manifest connections between obesity and obesity-related diseases

SUPPLEMENTARY MATERIALS

Supplementary Codes

All R codes used in this study are downloadable from http://pan.baidu.com/s/1kUO0lMZ with password '9ssn'.

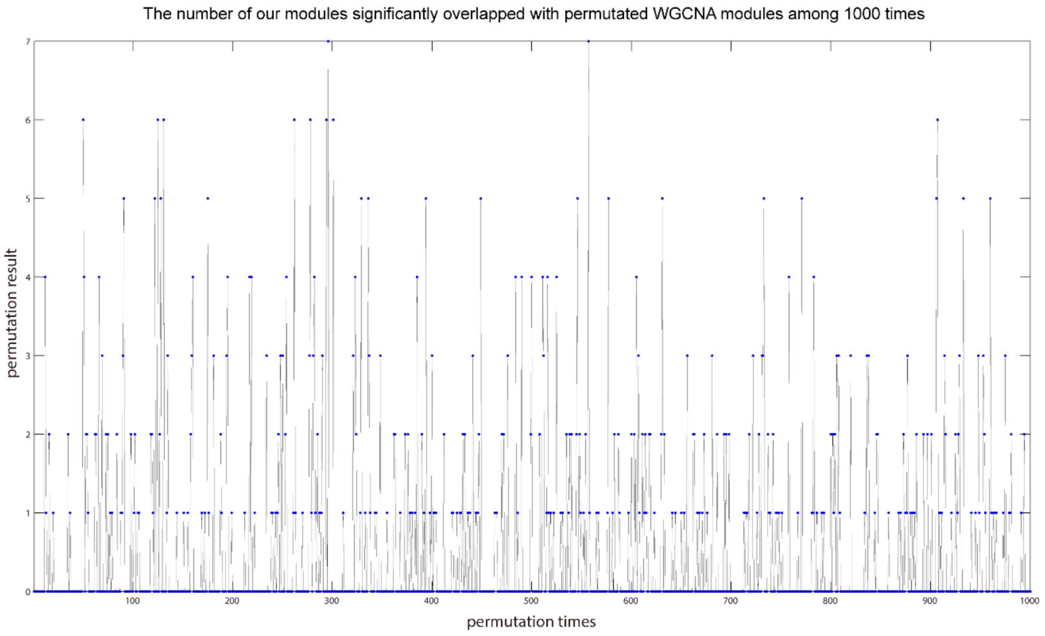

Supplementary Figure 1: Numbers of significant overlapped OBNet modules with WGCNA modules in the 1000 permutation runs.

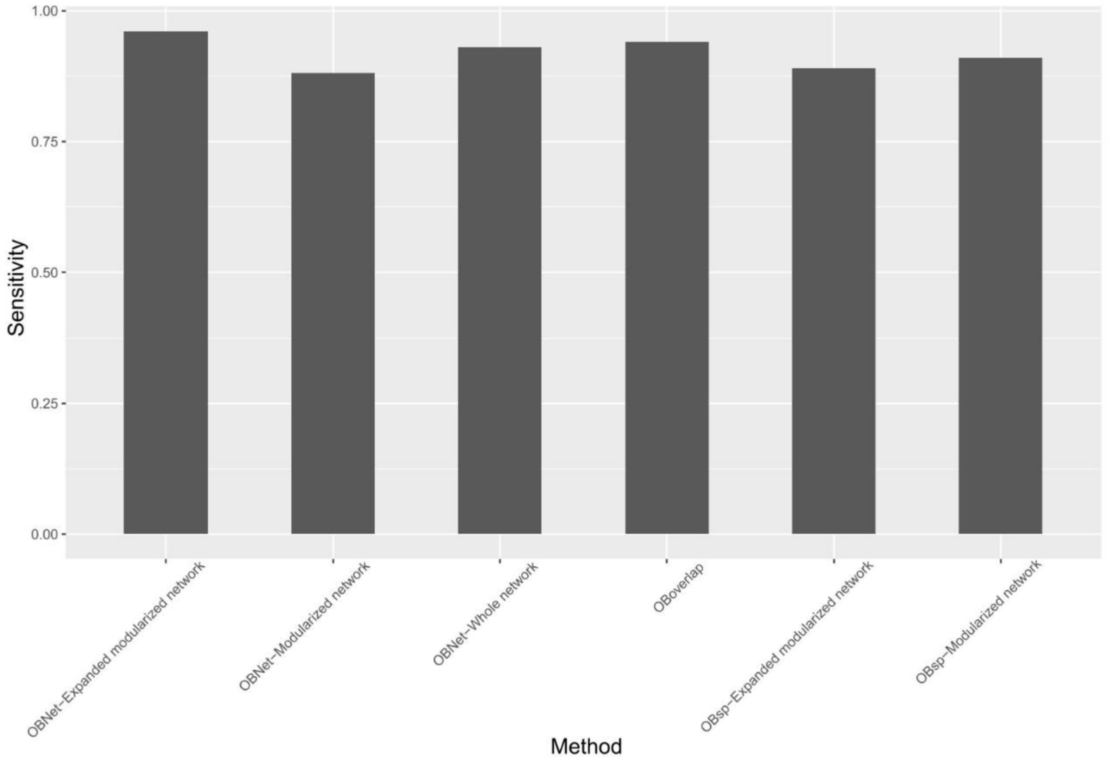

Supplementary Figure 2: Comparison of OBNet, OBsp and OBoverlap by sensitivity. OBNet-Expanded modularized network represents OBNet using expanded modularized network; OBNet-modularized network represents OBNet using modularized network; other methods are defined similarly.

Supplementary Table 1: Obesity-related diseases by literature mining

Disease name	Jaccard coefficient	Disease name	Jaccard coefficient
Obesity	1	Heart failure	0.009175
Type 2 diabetes	0.084545	Breast cancer	0.007515
Adiposity	0.062313	Pulmonary function	0.007479
Cardiovascular disease risk factors	0.055003	Osteoarthritis	0.006777
Sjörgren’s syndrome	0.045295	Type 1 diabetes	0.006632
Blood pressure	0.044218	Bone mineral density	0.006393
Height	0.043781	Colorectal cancer	0.005506
Visceral fat	0.032821	Insulin-like growth factors	0.005356
Adiponectin levels	0.021645	Tumor biomarkers	0.00534
Smoking behavior	0.0216	Carotid intima media thickness	0.00532
C-reactive protein	0.020601	Intelligence	0.005108
Insulin resistance/response	0.018967	Cardiac hypertrophy	0.00504
Waist-hip ratio	0.018877	Preeclampsia	0.004666
Ageing	0.018483	Chronic obstructive pulmonary disease	0.004551
Birth weight	0.018034	Prostate cancer	0.004504
Eating disorders	0.014978	Metabolic traits	0.004365
Nephropathy	0.013048	Endometrial cancer	0.004115
Body mass index (interaction)	0.011998	Schizophrenia	0.003774
Coronary artery disease	0.011556	Major depressive disorder	0.003753
Polycystic ovary syndrome	0.011454	Alzheimer’s disease	0.003326
Heart rate	0.011083	Inflammatory bowel disease	0.003148
Chronic kidney disease	0.01064	Atrial fibrillation	0.003007
Educational attainment	0.010369	Rheumatoid arthritis	0.002485
Asthma	0.009872	Coronary artery calcification	0.000922
Brain structure	0.009629	Type 1 diabetes nephropathy	0.000824
Inflammatory biomarkers	0.009561		

Supplementary Table 2: The association of obesity and 147 diseases by OBNet-Expanded modularized network.

See Supplementary File 1

Supplementary Table 3: Detailed information about the top 10 differential modules inferred by WGCNA

Module	MDC	MDC-FDR	Gene number	Function annotation	Function annotation-FDR
blue	1.47	0	1488	GO:0006955~immune response	8.11E-128
darkgreen	2.46	0	79	GO:0009615~response to virus	3.65E-22
darkorange	2.28	0	67	hsa05310:Asthma	3.64E-11
darkgrey	1.04	0	70	GO:0010942~positive regulation of cell death	5.70E-08
royalblue	1.46	0	104	GO:0050851~antigen receptor-mediated signaling pathway	1.73E-05
lightgreen	1.29	0	111	GO:0030003~cellular cation homeostasis	6.58E-04
orange	1.8	0	67	GO:0065004~protein-DNA complex assembly	2.24E-55
white	2.47	0	66	GO:0043368~positive T cell selection	4.89E-03
saddlebrown	2.25	0	40	GO:0015630~microtubule cytoskeleton	0.07
skyblue	3.1	0	48	IPR019775:WD40 repeat, conserved site	3.68E-04

Supplementary Table 4: The top 10 modules mediating the interaction between breast cancer and obesity

Module	Disease	p-Value	FDR
GO:0030902_hindbrain development	Breast cancer	2.03E-05	1.74E-03
GO:0033135_regulation of peptidyl-serine phosphorylation	Breast cancer	3.69E-05	2.69E-03
GO:0021695_cerebellar cortex development	Breast cancer	1.80E-04	9.15E-03
GO:0030003_cellular cation homeostasis	Breast cancer	3.82E-04	1.66E-02
GO:0022037_metencephalon development	Breast cancer	3.98E-04	1.71E-02
GO:0006521_regulation of cellular amino acid metabolic process	Breast cancer	6.44E-04	2.44E-02
GO:0048871_multicellular organismal homeostasis	Breast cancer	7.00E-04	2.58E-02
GO:0055080_cation homeostasis	Breast cancer	7.46E-04	2.71E-02
GO:0055082_cellular chemical homeostasis	Breast cancer	9.37E-04	3.21E-02
GO:0031145_anaphase-promoting complex-dependent proteasomal ubiquitin-dependent protein catabolic process	Breast cancer	1.04E-03	3.48E-02

Supplementary Dataset 1: The obesity and disease associated genes used in this study.

See Supplementary Dataset 1

Supplementary Dataset 2: Association between obesity and diseases by literature mining.

See Supplementary Dataset 2

Supplementary Dataset 3: The literatures supporting the 51 obesity-related diseases.

See Supplementary Dataset 3

Supplementary Dataset 4: Significance of obesity-disease association by OBNet.

See Supplementary Dataset 4

Supplementary Dataset 5: Genes associated to 29 WGCNA modules.

See Supplementary Dataset 5
