# Supplementary material for: Using molecular functional networks to manifest connections between obesity and obesity-related diseases
Source: Oncotarget. 2017 Jul 22;8(49):85136–49. doi: 10.18632/oncotarget.19490 (PMC5689599; doi:10.18632/oncotarget.19490)
Supplement: Supplementary file 2 [file oncotarget-08-85136-s002.doc]

**Supplementary Table 2:** **The association of obesity and 147 diseases by OBNet-Expanded modularized network**

**DiseaseFDRDiseaseFDR**Body mass index3.15E-32Primary biliary cirrhosis1.30E-03Autism spectrum disorder-bipolar disorder-schizophrenia7.60E-11Chronic obstructive pulmonary disease1.39E-03Coronary artery disease3.29E-09Systemic sclerosis1.41E-03Type 2 diabetes3.47E-09Menarche (age at onset)1.44E-03Metabolite levels7.60E-09Chronic kidney disease1.64E-03Atrial fibrillation8.76E-09Liver enzyme levels (alkaline phosphatase)1.67E-03Height9.42E-09Lipoprotein-associated phospholipase A2 activity and mass1.87E-03Obesity-related traits5.26E-08Multiple sclerosis (OCB status)2.16E-03Chronic lymphocytic leukemia5.51E-08White blood cell count2.20E-03Type 1 diabetes6.71E-08Asthma and hay fever2.23E-03Bone mineral density9.23E-08Celiac disease and Rheumatoid arthritis2.55E-03Blood trace element (Cu levels)1.34E-07Paget's disease3.12E-03Neuroblastoma1.34E-07IgE levels3.25E-03Warfarin maintenance dose1.34E-07Hypertriglyceridemia3.34E-03C-reactive protein1.36E-07Activated partial thromboplastin time4.02E-03Metabolic syndrome1.47E-07Migraine4.28E-03HDL cholesterol1.77E-07Chronic obstructive pulmonary disease-related biomarkers5.48E-03Alzheimer's disease2.21E-07Alopecia areata5.85E-03Thyroid function2.24E-07Progressive supranuclear palsy5.88E-03Triglycerides6.69E-07Male-pattern baldness5.98E-03Crohn's disease7.96E-07Thyroid cancer7.41E-03Asthma8.48E-07Aortic root size7.49E-03Red blood cell traits1.05E-06Glycemic traits (pregnancy)7.76E-03Prostate cancer1.16E-06Atopic dermatitis9.59E-03Fasting plasma glucose1.68E-06Hepatitis B1.04E-02Celiac disease1.70E-06Hypertension1.15E-02Blood pressure2.25E-06Homocysteine levels1.18E-02Rheumatoid arthritis2.50E-06Epstein-Barr virus immune response (EBNA-1)1.44E-02Inflammatory biomarkers4.07E-06Erythrocyte sedimentation rate1.44E-02HDL Cholesterol - Triglycerides (HDLC-TG)4.73E-06Multiple myeloma1.52E-02Lipid traits1.25E-05White blood cell types1.53E-02Platelet counts1.85E-05Phosphorus levels1.90E-02Allergic sensitization1.90E-05C-reactive protein levels2.37E-02Pulmonary function1.94E-05Acute lymphoblastic leukemia2.79E-02Breast cancer2.03E-05IgG glycosylation2.83E-02Electrocardiographic traits2.38E-05Lymphoma2.88E-02Inflammatory bowel disease2.87E-05Nasopharyngeal carcinoma3.33E-02Schizophrenia or bipolar disorder2.88E-05Sj?gren's?Syndrome3.47E-02Immune response to smallpox vaccine (IL-6)3.34E-05Phospholipid levels (plasma)4.06E-02Fibrinogen3.46E-05Mean corpuscular hemoglobin concentration4.96E-02Weight4.01E-05Hematology traits5.29E-02Age-related macular degeneration4.24E-05Psoriasis5.72E-02Endometriosis4.40E-05Sphingolipid levels6.89E-02Immune reponse to smallpox (secreted IL-2)6.08E-05Alcohol dependence7.32E-02Heart rate6.12E-05Amyotrophic lateral sclerosis8.18E-02Testicular germ cell cancer6.19E-05Dupuytren's disease8.95E-02Colorectal cancer6.39E-05Non-obstructive azoospermia9.99E-02Systemic lupus erythematosus and Systemic sclerosis7.00E-05Polycystic ovary syndrome1.12E-01Nephropathy7.60E-05Primary tooth development1.19E-01Behcet's disease7.98E-05Abdominal aortic aneurysm1.32E-01Corneal structure8.97E-05Smoking behavior1.35E-01Hypothyroidism9.09E-05Palmitoleic acid (16:1n-7) plasma levels1.95E-01Pancreatitis1.22E-04Helicobacter pylori serologic status2.20E-01Lipid metabolism phenotypes1.25E-04Myocardial infarction (early onset)2.24E-01Parkinson's disease1.26E-04Amyloid A serum levels2.72E-01Uric acid levels1.36E-04Menarche and menopause (age at onset)2.80E-01Complement C3 and C4 levels1.66E-04Bladder cancer3.68E-01Glaucoma1.78E-04Bilirubin levels4.17E-01Glycated hemoglobin levels2.02E-04Serum metabolite levels4.36E-01Refractive error2.94E-04Metabolic syndrome (bivariate traits)4.77E-01Carotid intima media thickness3.16E-04Liver enzyme levels5.15E-01Hematological and biochemical traits3.55E-04acid (18:0) plasma levels5.22E-01Ulcerative colitis3.84E-04Plasma omega-6 polyunsaturated fatty acid levels6.13E-01Cardiovascular disease risk factors3.85E-04Protein quantitative trait loci6.30E-01Graves' disease4.38E-04Kawasaki disease6.41E-01Liver enzyme levels (gamma-glutamyl transferase)4.59E-04Iron status biomarkers6.49E-01Retinal arteriolar caliber5.09E-04Hair color6.62E-01Adiponectin levels6.43E-04Coagulation factor levels6.75E-01Lung cancer7.43E-04Basal cell carcinoma7.19E-01Breast size8.02E-04Axial length7.62E-01Melanoma8.13E-04Ovarian cancer7.76E-01Vitiligo1.01E-03Beta-2 microglubulin plasma levels8.52E-01Hodgkin's lymphoma1.18E-03Antineutrophil cytoplasmic antibody-associated vasculitis8.77E-01Multiple sclerosis1.23E-03
